# Plague Circulation and Population Genetics of the Reservoir *Rattus rattus*: The Influence of Topographic Relief on the Distribution of the Disease within the Madagascan Focus

**DOI:** 10.1371/journal.pntd.0002266

**Published:** 2013-06-06

**Authors:** Carine Brouat, Soanandrasana Rahelinirina, Anne Loiseau, Lila Rahalison, Minoariso Rajerison, Dominique Laffly, Pascal Handschumacher, Jean-Marc Duplantier

**Affiliations:** 1 IRD, UMR022 CBGP, Montferrier-sur-Lez, France; 2 Institut Pasteur de Madagascar, Antananarivo, Madagascar; 3 INRA, UMR1062 CBGP, Montferrier-sur-Lez, France; 4 Département de Géographie, UFR Sciences Espaces Sociétés, Université de Toulouse, Toulouse, France; 5 GEODE UMR 5602 CNRS, Toulouse, France; 6 IRD, UMR912 SE4S, Marseille, France; University of Tennessee, United States of America

## Abstract

**Background:**

Landscape may affect the distribution of infectious diseases by influencing the population density and dispersal of hosts and vectors. Plague (*Yersinia pestis* infection) is a highly virulent, re-emerging disease, the ecology of which has been scarcely studied in Africa. Human seroprevalence data for the major plague focus of Madagascar suggest that plague spreads heterogeneously across the landscape as a function of the relief. Plague is primarily a disease of rodents. We therefore investigated the relationship between disease distribution and the population genetic structure of the black rat, *Rattus rattus*, the main reservoir of plague in Madagascar.

**Methodology/Principal Findings:**

We conducted a comparative study of plague seroprevalence and genetic structure (15 microsatellite markers) in rat populations from four geographic areas differing in topology, each covering about 150–200 km^2^ within the Madagascan plague focus. The seroprevalence levels in the rat populations mimicked those previously reported for humans. As expected, rat populations clearly displayed a more marked genetic structure with increasing relief. However, the relationship between seroprevalence data and genetic structure differs between areas, suggesting that plague distribution is not related everywhere to the effective dispersal of rats.

**Conclusions/Significance:**

Genetic diversity estimates suggested that plague epizootics had only a weak impact on rat population sizes. In the highlands of Madagascar, plague dissemination cannot be accounted for solely by the effective dispersal of the reservoir. Human social activities may also be involved in spreading the disease in rat and human populations.

## Author Summary

Plague (*Yersinia pestis* infection) is a highly virulent disease occurring in many natural ecosystems. One of the major plague foci is Madagascar, where plague has been confined principally to the central highlands since the 1920s. Despite the high prevalence of plague, its ecology has scarcely been studied. Previous studies of plague seroprevalence in humans have suggested that the disease spreads unevenly across landscapes, possibly due to the relief. Plague is primarily a disease of rodents. We therefore investigated the relationship between its distribution and the population genetic structure of its main reservoir, the black rat, *Rattus rattus*. We used serological and microsatellite-based population genetic analyses to compare four geographic areas of the plague focus (two mountainous areas, two plateaus). Rat populations had a more marked genetic structure in areas of greater relief. The heterogeneous distribution of plague in the highlands may be related to the population genetic structure of its reservoir, but our results suggest also a role for humans in the dispersion of the disease.

## Introduction

Evaluating the contribution of landscape characteristics to spatial heterogeneity in the occurrence of infectious diseases is critical for the assessment of risks and the design of optimal surveillance and control programs. Plague (*Yersinia pestis* infection) is a highly virulent, re-emerging disease that has caused three pandemics in the last 2000 years [1]. Several hundred human cases are reported each year, mostly in Africa ([2]). Despite its major impact on human populations, plague is primarily a disease of rodents and the fleas associated with them. Plague ecology has been extensively studied in North America, which accounts for only 1.2% of all recent cases in humans [2]. In this region, landscape structure accounted for the spread of the disease [3], [4] through effects on the dispersal of the rodent host [5]. For instance, the occurrence of plague was found to be negatively associated with the presence of putative dispersal barriers, such as rivers, streams or roads, within the landscape [3]. Furthermore, the occurrence of an unfavorable habitat for reservoirs was associated with significant genetic structure in populations of the plague bacterium, even when the geographic distance between trap sites was small [4]. In major plague foci (such as the Democratic Republic of the Congo and Madagascar, which together account for 90.1% of all human cases worldwide [2]), plague ecology has been little studied. However, African plague foci may differ considerably from American foci, because the reservoir or vector species involved may have very different ecological features and distributions [6].

Plague was introduced into Madagascar in 1898, and has remained endemic in the central highlands of the country since the 1920s, with hundreds of human cases reported each year [7] and continuous circulation within rodent populations [8]. Human seroprevalence levels and reported plague cases are heterogeneously distributed across the highlands of Madagascar [7], [9], [10]. It has been suggested that this spatial variability in the occurrence of the disease within human populations is related to topographic relief (e.g., the degree of change in elevation within a particular geographic area). Indeed, a comparative analysis of two adjacent areas showed that villages in which positive plague seroprevalence was found in humans tended to be clustered together in the mountainous area, but more evenly distributed across the landscape in the plateau area [11]. This pattern may reflect differences in the degree of human movements and exchanges, and hence, in the translocation of infected rodents or fleas between villages. Alternatively, as shown for other biological systems (e.g., [12]), it may reflect effects of the landscape on the population genetic structure of disease vectors and/or reservoirs (e.g. fleas and rodents).

The genetic structure of flea populations is thought to be strongly determined by host dispersal patterns ([13], but see [14], [15]). In Madagascar, two fleas, *Xenopsylla cheopis* and *Synopsyllus fonquerniei*, have been identified as plague vectors [8]. Both species are known to be specific parasites of rodents and insectivores [16]. In rural areas of the highlands, their principal host is the black rat, *Rattus rattus*, which is also the main reservoir of plague [8], [17]. This rodent species was introduced by humans several centuries before the arrival of the disease on this island [18]. It accounts for more than 95% of all small mammal captures in the highlands of Madagascar (except in isolated forest fragments and towns) [19], suggesting that other reservoir species are unlikely to play a major role in plague spread. *Rattus rattus* is known to have a limited home range and low levels of dispersal (e.g., a few hundred meters, although much larger distances of a few kilometers, have been observed on occasion) [20], [21], [22], with reported population densities of a few tens of individuals per hectare [23], [24]. The few ecological data available for this species suggested that genetic differentiation may occur over a fine spatial scale, and that geographic barriers may influence gene flow and population structure [25].

Topographic relief has been shown to influence gene flow in many species (see refs. in [26]) including rodents [27]. We investigated the role of rat dispersal in plague spread, by carrying out a comparative study of plague seroprevalence levels and the genetic structure of rat populations in four areas of the Madagascan plague focus with different topographic relief profiles. We aims at answering the following questions: (1) Does the distribution of plague seroprevalence in rat populations differs between plateau and mountain areas, as shown in humans [11]?; (2) Is the fine-scale genetic structure of rat populations higher in mountain areas than in plateaus, suggesting an effect of landscape (more highly structured by rivers and ridges in mountains than on plateaus) on rat dispersal?; and (3) how is plague distribution related to population genetics of *R. rattus*?

## Materials and Methods

### Ethics statement

Field sampling was carried out systematically by joint teams of staff from the IRD (Institut de Recherche pour le Développement), the IPM (Institut Pasteur de Madagascar) and the Madagascan Ministry of Health (Communicable Disease Control Department). Each trapping campaign was validated by the national, regional and local health authorities. Traps were set within houses with the approval of the owner or tenant. Outside houses, traps were set with the agreement of the village head, and always on the edge of cultivated fields, so as not to damage crops. The only rodents sampled were those from introduced species (house mouse, black rat and Norway rat), all of which are considered as pests, with no protected status in Madagascar. No permission was therefore required for their capture.

Animals were treated in a humane manner, in accordance with the guidelines of the American Society of Mammalogists [28]. Madagascar has no ethics committee overseeing animal experimentation, and the IRD has no ethics board for the review of animal experimentation protocols. However, the ANR-SEST (Agence Nationale pour la Recherche, Santé-Environnement et Santé-Travail) program on plague diffusion, which provided some of the funding for this project, has been approved by the Managing Director of the IRD. In addition, regional approval was obtained from the regional Head of Veterinary Service (Hérault, France), for the sampling and killing of rodents and the harvesting of their tissues (approval no. B 34-169-1) carried out during this study.

### Study areas and sample collection

**Figure 1:**
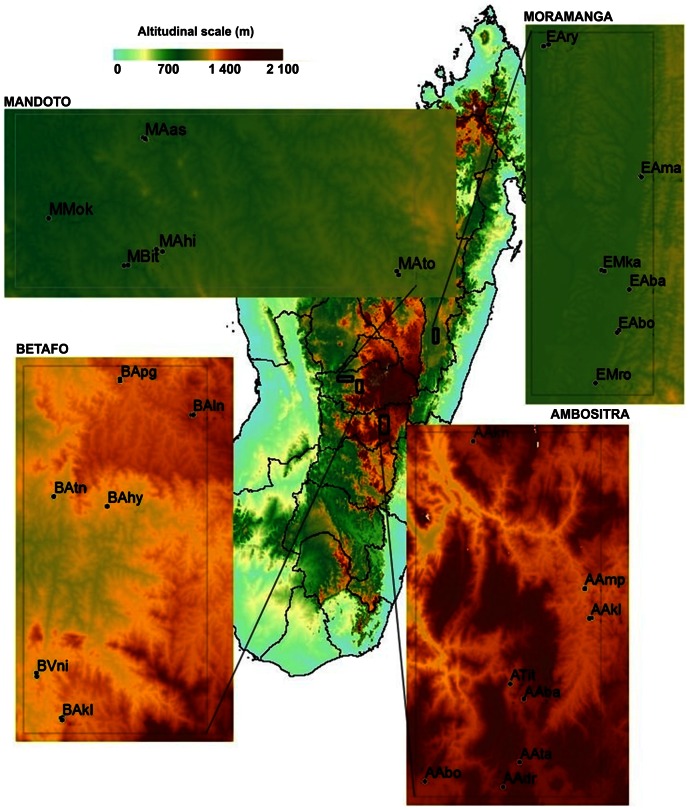
Geographic location of the four areas studied and their sampled subpopulations in Madagascar. Mandoto, Moramanga: plateau areas; Betafo, Ambositra: mountainous areas.

Four study areas in which human plague epidemics are regularly reported were compared (Figure 1, Table 1). Moramanga is an area located in a wide valley, along the Mangoro River, with a slight decrease in elevation along a gradient running from north to south. Mandoto is a plateau area with rolling hills and a fairly flat relief. Rat samples from this area have already been used in a population genetic study [29], but we used 10 additional markers in this study (see below). In Betafo, the landscape is more rugged, with relatively large changes in elevation between villages. Ambositra is a mountainous area, in which villages are located in deep valleys separated by high ridges. Mandoto and Betafo have been studied before, in investigations of the distribution of plague seroprevalence in humans [11].

**Table 1:** Study areas and their characteristics.

	Moramanga	Mandoto	Betafo	Ambositra
Landscape	Plateau	Plateau	Relief	Relief
Sampling date	Apr. 2008–Nov. 2009	Sept. 1996–Jan. 1997	Sept. 2006–Jan. 2007	Feb.–Apr. 2000
Village number	6	5	6	8
Subpopulation number	10	9	10	11
Number of sampled/genotyped rats	188/179	464/262	386/275	269/194
Min/Max D_E_	2.6/28.6	1.9/20.9	3.7/26.3	3.2/35.8

D_E_ = Euclidean distance among villages (km).

Sampling was performed in 1996–1997 (Mandoto), 2000 (Ambositra), 2006–2007 (Betafo) and 2008–2009 (Moramanga), in six to eight villages per area (Table 1). Villages were at least 2 km apart (separated by a maximum of 36 km), to ensure that they were further apart than the distance generally covered by rats around their territory (a few hundred meters, e.g. [20], [21], [22]). Within each village, we sampled two trap sites corresponding to different habitats, as described in a previous protocol [29]: i) within the villages, in houses and sisal fences around the enclosures close to the houses (V), and ii) in the surrounding irrigated rice fields and crops (R) a few hundred meters from the houses. This sampling scheme was chosen because previous studies have shown that rat subpopulations from these habitats differ in term of their relative densities, the duration of the breeding season (continuous within villages, restricted to the warm season in fields), their flea species (*X. cheopis* on the rats from houses, *S. fonquerniei* on the rats from fields) and flea abundance (higher on the rats from houses), and that the movements of rats between habitats are limited [22], [29]. This made it possible for us to evaluate the contribution of such differences to genetic structure at a fine spatial scale. Trap sites were geo-referenced with a Global Positioning System (GPS).

Each captured rat was euthanized by cervical dislocation, then weighted, measured and autopsied. Blood was collected from the heart on seropads (Laboratoire de Développement et d'Analyses 22440; LDA22 Zoopole–Ploufragan), and a piece of ear or tail was stored in ethanol for molecular analyses.

### Serological diagnosis of plague

For each sampled rat, specific antibodies against *Y. pestis* were detected in seropads by a rat IgG anti-F1 enzyme-linked immunosorbent assay (sensitivity of 100%; specificity of 98.7% for an optical density threshold of 0.05) [30]. Plague seroprevalence was then calculated as the percentage of seropositive individuals per trap site, taking all the rats analyzed into account.

### Molecular methods

We carried out molecular analyses on a maximum of 44 rats per trap site, the rats from a given trap site being considered as a subpopulation (Table 2). Genomic DNA was extracted from ethanol-stored tissues with the puregene dna purification kit. Genotyping was conducted for 15 microsatellite loci. Six of these loci were originally developed for *Rattus norvegicus* (D7Rat13, D10Rat20, D11Mgh5, D11Rat56, and D16Rat81; [31]). Samples from Mandoto had already been genotyped for these markers [29], but some were re-analyzed for the purposes of comparison. Ten additional microsatellite loci were recently developed specifically for *R. rattus* (Rr14, Rr17, Rr21, Rr22, Rr54, Rr67, Rr68, Rr93, Rr107, and Rr114: [32]), and were genotyped for the entire dataset. Every individual successfully genotyped at some loci but not at some others underwent re-amplification once by simple PCR (to avoid primer competition), for each failed locus.

**Table 2:** Seroprevalence and genetic estimates per subpopulation.

Area	Subpop	SP (%)	N	*H_S_*	*r*	*F_IS_*	*N_E_*	*F_ST_*
Moramanga	EAboV	0	8	0.71	5.4	0.032	14 [9; 22]	
	EAboR	0	14	0.68	4.8	0.057	74 [34; ∞]	
	EMkaV	0	21	0.73	5.3	0.092	36 [28; 50]	
	EMkaR	0	30	0.75	5.4	−0.008	25 [21; 31]	
	EAmaV	0	21	0.73	5.4	0.039	42 [29; 70]	
	EAmaR	0	16	0.71	5.0	0.011	44 [28; 93]	
	EAryV	0	11	0.71	5.1	0	92 [36; ∞]	
	EAryR	0	22	0.75	5.4	0.065	71 [48;131]	
	EAbaR	0	22	0.72	5.3	0.044	395 [79; ∞]	
	EMroR	7.14	14	0.68	4.6	0.157*	125 [35; ∞]	
	**Mean**			**0.72±0.02**	**5.2±0.3**	**0.046 [0.018; 0.076]**	**91.9±111.6**	**0.019 [0.01; 0.027]**
Mandoto	MAasR	3.17	21	0.69	4.6	0.064	30 [21; 45]	
	MAasV	1.69	44	0.72	5.3	0.019	64 [51; 84]	
	MAhiR	12.50	16	0.75	5.6	0.041	/	
	MAhiV	34.88	44	0.70	5.2	0.116*	42 [31; 62]	
	MAtoR	9.09	22	0.68	4.7	0.005	30 [23; 42]	
	MAtoV	8.06	42	0.67	4.9	0.037	45 [36; 58]	
	MBitR	5.55	10	0.72	5.4	0.015	/	
	MBitV	3.30	44	0.73	5.1	0.063	37 [31; 45]	
	MMokV	8.69	19	0.70	5.1	0.025	31 [19; 67]	
	**Mean**			**0.70±0.03**	**5.0±0.4**	**0.049 [0.004; 0.101]**	**39.9±12.2**	**0.038 [0.029; 0.047]**
Betafo	BAklV	0	36	0.73	5.2	0.032	39 [31; 51]	
	BAklR	0	20	0.67	4.7	−0.052	14 [11; 17]	
	BVniV	0	40	0.68	5.0	0.06	40 [32; 53]	
	BVniR	0	20	0.67	4.6	−0.16	23 [17; 33]	
	BAlnV	11.32	40	0.68	5.0	0.066	51 [41; 67]	
	BAlnR	0	15	0.64	4.5	−0.039	24 [16; 42]	
	BApgV	12.96	40	0.70	5.0	0.067	28 [23; 34]	
	BApgR	33.33	15	0.66	4.6	0.04	16 [12; 22]	
	BAhyV	0	35	0.70	5.0	0.101*	52 [39; 77]	
	BAtnV	45.45	14	0.72	5.2	0.104	373 [67; ∞]	
	**Mean**			**0.68±0.03**	**4.9±0.3**	**0.046 [−0.001; 0.011]**	**66.1±108.7**	**0.041 [0.033; 0.050]**
Ambositra	AAboV	0	22	0.75	5.5	0.042	33 [24; 47]	
	AAdrV	0	22	0.71	5.1	0.058	58 [32; 191]	
	AAtaV	0	22	0.73	5.1	0.085	82 [48; 231]	
	AAbaV	0	16	0.69	4.7	0.148*	17 [13; 23]	
	ATitR	0	17	0.56	3.7	−0.121	5 [3; 6]	
	AAklV	0	10	0.71	4.8	−0.028	8 [6; 12]	
	AAklR	0	9	0.71	4.5	0.01	11 [7; 19]	
	AAknV	0	29	0.73	5.3	0.052	19 [16; 22]	
	AAknR	0	11	0.72	4.8	−0.014	19 [13; 34]	
	AAmpV	5.56	22	0.68	4.6	0.032	9 [7; 11]	
	AAmpR	0	14	0.71	5.2	0.043	21 [14; 34]	
	**Mean**			**0.70±0.05**	**4.8±0.5**	**0.038 [−0.001; 0.093]**	**25.6±23.9**	**0.053 [0.045; 0.064]**

Seroprevalence (SP) was calculated on all sampled individuals per subpopulation, and genetic estimates were calculated on genotyped individuals (N) per subpopulation. Mean and standard errors (*r, H_S_*) or 95% confidence intervals (*F_IS_, F_ST_*) are reported for each area.

* indicated significant *F_IS_* values after correction for multiple tests.

### Data analyses

Deviations from Hardy–Weinberg equilibrium (HWE) within subpopulations and genotypic linkage disequilibrium (LD) between pairs of loci were tested using the Markov chain method implemented in Genepop v.4 [33]. We corrected for multiple testing by the False Discovery Rate (FDR) approach [34] implemented in the Qvalue package of R.

Some loci displayed significant heterozygote deficiencies in several subpopulations (see Results). In these loci, some null genotypes were found. We used Micro-checker v.2.2.3 [35] to test whether heterozygote deficiencies could be accounted for by the existence of null alleles. We then used Freena ([36], available from www.montpellier.inra.fr/URLB), to assess the need to correct for null alleles.

The genetic diversity of each subpopulation was assessed by calculating Nei's unbiased genetic diversity (H_S_, [37]), allelic richness (*r*, evaluating the number of alleles independent of sample size, calculated for a minimum of 7 individuals by the rarefaction procedure [38] implemented in Fstat v.2.9.3.2 [39]), and *F_IS_* (tested for significance with Fstat). Effective population size (*N_E_*) was estimated with Ldne [40]. The method used is based on linkage disequilibrium and assumes random associations of alleles at different loci. Alleles with a frequency ≥0.02 were used to minimize possible bias [40]. We investigated whether plague epizootics had left detectable traces on genetic diversity and population size, by assessing the relationship between *r*, *H_S_*, *F_IS_* or *N_E_* and plague seroprevalence in each subpopulation within areas by carrying out Spearman's rank non parametric correlation analyses in SAS v. 9.3 [41]. We compared *r*, *H_S_*, and *F_IS_* between areas with Fstat (10,000 permutations), and *N_E_* using nonparametric Kruskal-Wallis tests in SAS.

Genetic structure was examined in several ways within the four study areas. Bayesian clustering analyses were first performed with Structure v.2.3.3 [42]. This approach is based on an explicit evolutionary model for genetic variation and makes statistical inference on the basis of individual data, to estimate the number of genetic clusters in each area and assign individuals to the various clusters. We used the ΔK method to infer the number of genetic groups per area [43]. All analyses were performed with an admixture model and correlated allele frequencies [44]. We performed 10 independent runs for each K value (from 1 to *n*+1, *n* being the number of villages sampled in each area). Each run included 50,000 burn-in iterations followed by 500,000 iterations. We also checked that a single mode was obtained in the results of the 10 Structure runs for each K value, by using the Greedy algorithm implemented in Clumpp v.1.1.2 [45].

We also estimated *F_ST_* values [46] for each pair of subpopulations within each area, using Fstat. We generated 95% confidence intervals (CIs) for the mean *F_ST_* per area by bootstrap resampling across loci, and we then used Fstat (10,000 repetitions) to compare mean *F_ST_*. We assessed whether villages may explain population genetic structure within a given area, by performing AMOVA (analysis of molecular variance, [47]) with Arlequin v.2.000 [48], using the locus-by-locus option. The variance components were tested using randomization (1,000 permutations). At the finest spatial scale, we used G-based (log-likelihood ratio) randomization tests [49] to evaluate the effect of habitat on genetic structure, with Fstat. These analyses were carried out with pairs of subpopulations corresponding to different habitats within villages (Betafo: 4 pairs; Mandoto: 4 pairs; Ambositra: 3 pairs; Moramanga: 4 pairs). Independent tests of pairwise genetic differentiation were combined, by the generalized binomial procedure implemented in Multitest v.1-2 [50].

We analyzed isolation by distance (IBD) by regressing pairwise estimates of *F*_ST_/(1 - *F*_ST_) against the logarithm of the Euclidean geographic distances between trap sites ([51]). Under a model of isolation by distance, genetic distance between subpopulations would be expected to increase with geographic distance. Mantel tests were performed to test the correlation between matrices of genetic differentiation and geographic distance in Genepop (10,000 permutations), excluding intra-village comparisons. The spatial pattern of genetic variation was also investigated by spatial autocorrelation analyses of mean genetic relatedness between pairs of individuals. These analyses complemented standard tests of isolation by distance, as spatial autocorrelation can occur at very fine scales, below the level of the area. Moreover, genetic relatedness provides a more contemporary picture of population genetic structure than the integrative *F_ST_*. Spatial autocorrelation analyses were performed for each area with Spagedi v.1.2 [52], and the relatedness coefficient *r_xy_* [53] was calculated for each distance class. Genotypic data for more than 398 pairs were included in each distance class. The null hypothesis of random genetic structure was rejected if the correlation coefficient exceeded the limits of the 95% confidence interval, as determined from 10,000 permutations.

Finally, we investigated the relationship between plague seroprevalence distribution and genetic structure in rat subpopulations while statistically controlling for the effect of the Euclidean geographic distance. This was done with partial Mantel tests [54] performed in Fstat (10,000 permutations), using pairwise absolute differences between seroprevalence levels and pairwise estimates of *F_ST_* between subpopulations. A positive correlation would suggest a strong influence of rat dispersal on plague distribution.

## Results

In total, we sampled 1297 rats in the four areas. In the mountain area of Ambositra and the plateau of Moramanga, most of the rats were seronegative, and plague seroprevalence levels were different from zero at only one trap site per area (Ambositra: AAmpV, two seropositive rats; Moramanga: EMroR, one seropositive rat) (Table 2). In Betafo, plague seroprevalence was positive only in the north westernmost villages (BAln, BApg, BAtn) (Figure 1; Table 2). In Mandoto, seropositive individuals were found in all villages (Table 2).

In total, 910 rats were genotyped. Ten loci (D10R20, D7R13, Rr14, Rr17, Rr22, Rr54, Rr68, Rr93, Rr107, Rr114) were at HWE. The other five (D11R56, D16R81, D11M5, Rr21, Rr67) displayed significant heterozygote deficiencies in several subpopulations, probably due to null alleles. Estimated null allele frequencies were low [36] (from 0.05 to 0.13; mean frequency for all loci = 0.037). All analyses yielded similar results with and without loci not at HWE: those presented here were obtained with the whole dataset. LD was significant for 25 of the 4200 tests performed (0.6% of comparisons), so the 15 loci were considered to be independent.

The 15 microsatellite loci considered in the analyses were polymorphic in all subpopulations (Table 2). In Ambositra and Moramanga, the subpopulations with positive plague seroprevalence also had low levels of genetic diversity and a high *F_IS_* (Table 2) Subpopulation-specific *N_E_* values were below 100 individuals, except in three subpopulations with infinite confidence intervals for which estimations were thus, by definition, imprecise (Table 2). Spearman's rank correlation analyses for genetic diversity and seroprevalence were carried out for Betafo and Mandoto (more than one subpopulation with seropositive rats). The only result close to statistical significance was a positive relationship between seroprevalence and *F_IS_* in Betafo (*r_s_* = 0.59, *P* = 0.07). In Mandoto, the subpopulation with the highest seroprevalence (MAhiV) also had the highest *F_IS_* (Table 2), but the relationship between these two factors was not significant. Genetic diversity did not differ between areas (*r*: *P* = 0.14; *H_S_*: *P* = 0.13; *F_IS_*: *P* = 0.96), unlike effective population size (*P* = 0.02), with the smallest *N_E_* values obtained for Ambositra and the highest ones for Moramanga.

**Figure 2:**
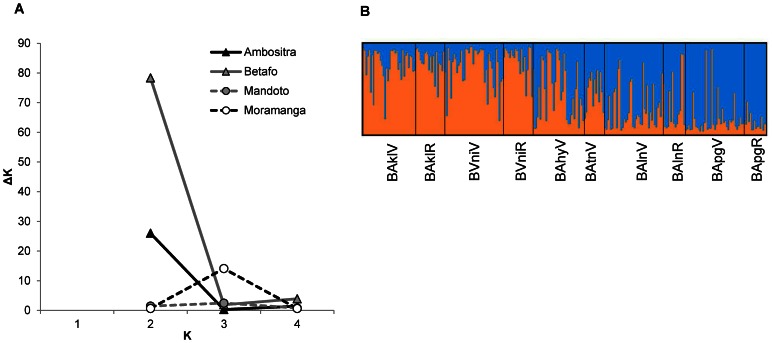
Results of Structure analyses. (A) Estimates of ΔK for each possible value of K within each of the four areas; (B) Structure bar plot for the run with the highest estimated posterior probability for Betafo at K = 2: subpopulations are ordered from south (left) to north (right).

Structure indicated the presence of a single genetic cluster in Moramanga (minor peak of ΔK not associated with an interpretable structure) and Mandoto (no maximum ΔK), and of two genetic clusters within the mountainous areas of Betafo and Ambositra (Figure 2A). In Betafo, the two genetic clusters clearly corresponded to northern and southern subpopulations (Figure 2B). In Ambositra, the clustering was weaker and was related to a pair of subpopulations, ATitR- AAmpV (proportion of membership to cluster 2: 0.92 for ATitR, 0.75 for AAmpV, <0.38 for other subpopulations). ATitR and AAmpV were not particularly similar in terms of their *F_ST_* and they are not located geographically close together (Euclidean distance of 13 km; Figure 1), but their genetic diversity was significantly lower than that of other subpopulations (Fstat permutation tests, *r*: *P* = 0.03; *H_S_*: *P* = 0.09; Table 2), suggesting possible artifactual clustering.

Overall *F_ST_* estimates differed between zones (*P* = 0.02), with the lowest value obtained for Moramanga and the highest for Ambositra (Table 2). Pairwise *F_ST_* estimates ranged from 0 to 0.05 in Moramanga, from 0.008 to 0.06 in Mandoto, from 0.01 to 0.09 in Betafo and from 0.02 to 0.16 in Ambositra. In all four areas, there was significant genetic differentiation between villages (Moramanga: *V_a_* = 0.88%, *P*<0.005; Mandoto: *V_a_* = 1.16%, *P*<0.002; Betafo: *V_a_* = 0.94%, *P*<0.003; Ambositra: *V_a_* = 1.17%, *P* = 0.02) although most of the genetic variation was observed within subpopulations (*V_c_*>94%, *P*<0.0001). There was also significant genetic differentiation between habitats within villages, in each area (unweighted mean *F_ST_*, Moramanga: *F_ST_* = 0.012; *P*<0.001; Mandoto: *F_ST_* = 0.024, *P*<0.001; Betafo: *F_ST_* = 0.037; *P*<0.001; Ambositra *F_ST_* = 0.053, generalized binomial test *P* = 0.003).

**Figure 3:**
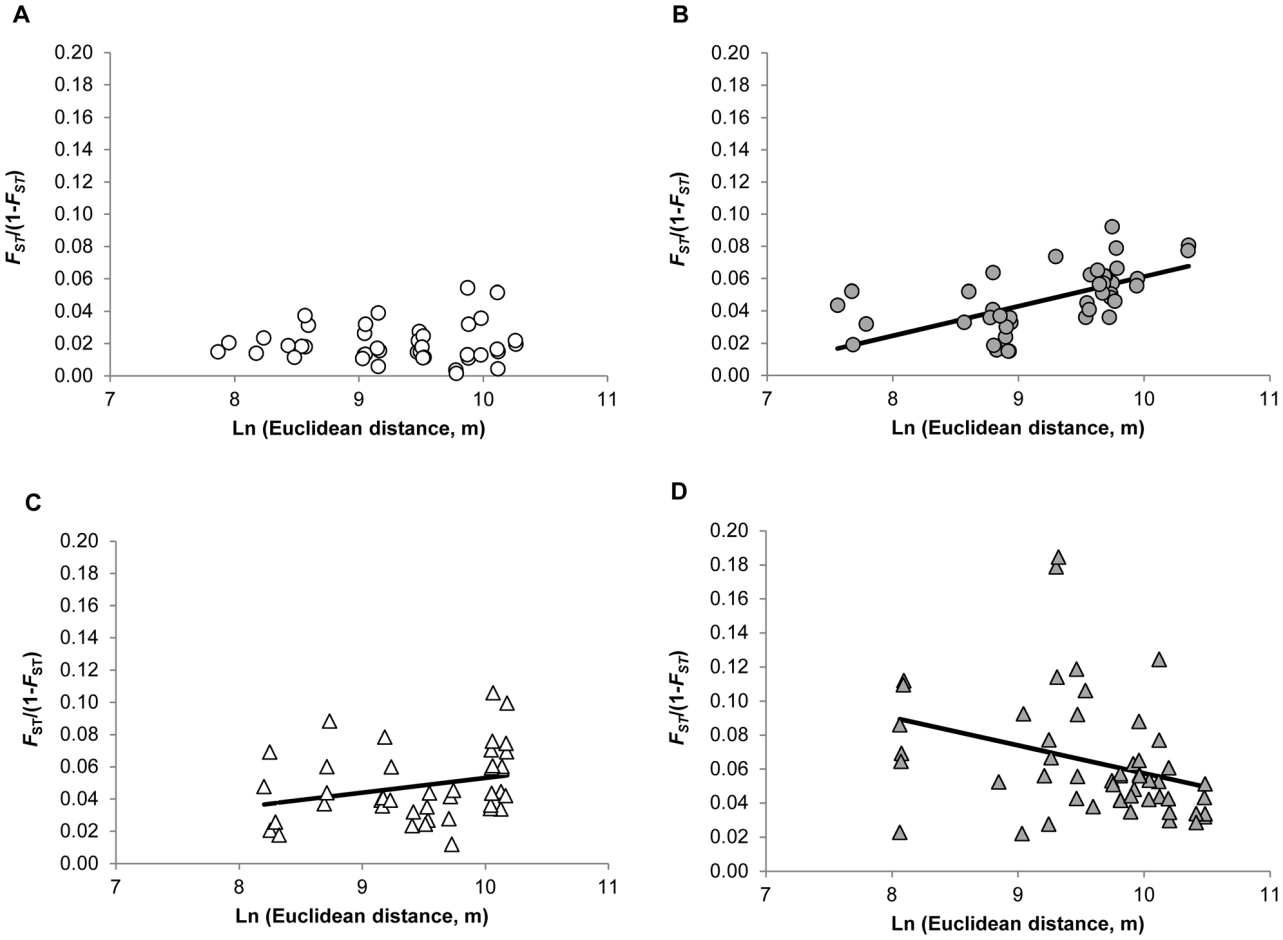
Genetic isolation by distance patterns in *Rattus rattus* populations of the four areas. Relationships (the regression line is shown only when significant) between pairwise Euclidean geographic distance between subpopulations and genetic differentiation, estimated as *F*_ST_/(1-*F*_ST_). (A) Moramanga, plateau area; (B) Mandoto, plateau area; (C) Betafo, mountainous area; (D) Ambositra, mountainous area.

Genetic IBD was significant in Betafo (*P* = 0.0001; slope *b* = 0.009, 95%CI = [0.0035; 0.022]) and in Mandoto (*P* = 0.02; slope *b* = 0.012, 95%CI = [0.005; 0.022]), but not in Moramanga (*P* = 0.59; slope *b* = 0.0004, 95%CI = [−0.005; 0.007]) (Figure 3). For Ambositra, genetic differentiation was negatively related to distance (*P* = 0.02; slope *b* = −0.016, 95%CI = [−0.02; −0.01]) (Figure 3D). However, this relationship was no longer significant (*P* = 0.09) if the ATitR/AAmpV subpopulations (which were clustered together in Structure analyses) were excluded.

The analysis of spatial genetic autocorrelation analysis yielded positive values for the first distance classes (within trap sites) in each area. For the second distance class (corresponding to distances between habitats within villages) correlation values were significant and positive only in Betafo and Mandoto. Genetic similarities between individuals subsequently decreased for higher-order classes, becoming mostly non-significant.

**Figure 4:**
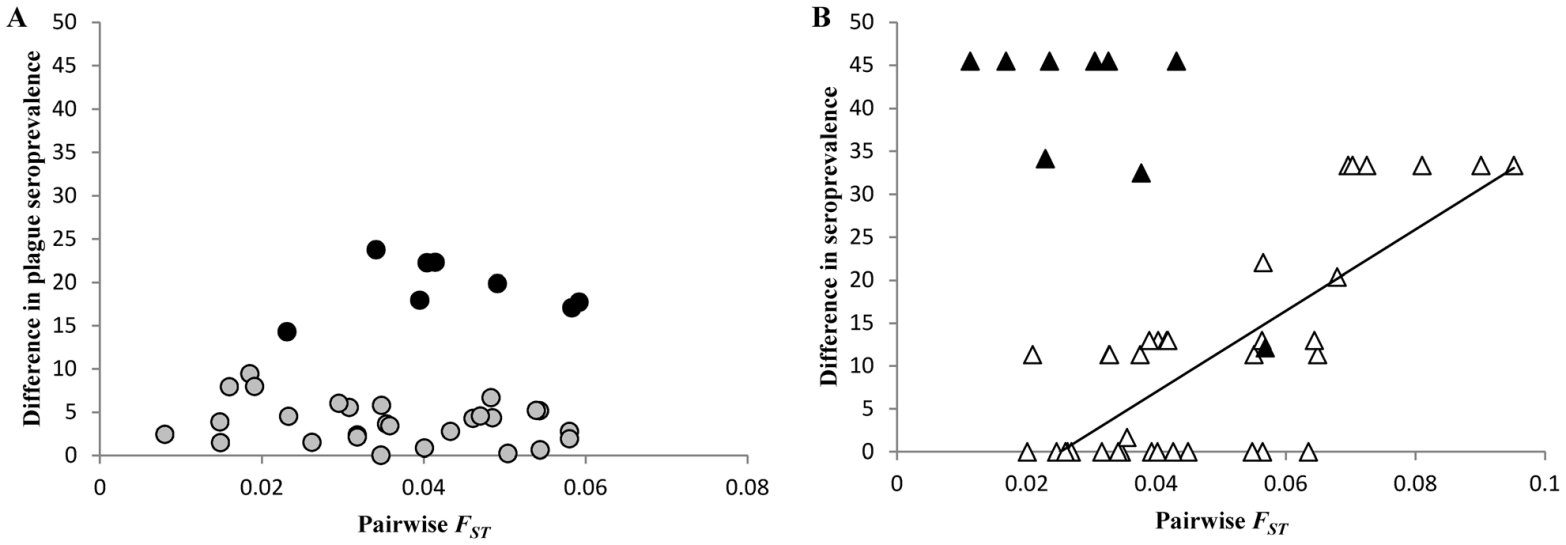
Relationship between plague seroprevalence data and genetic structure in rats. Each subpopulation pair was characterized by its absolute difference between seroprevalence levels, and its pairwise *F*_ST_. (A) Mandoto, plateau area: black circles indicated the subpopulations pairs involving MAhiV; (B) Betafo, mountainous area: black triangles indicated the subpopulation pairs involving BAtnV.

Partial Mantel tests using seroprevalence data were carried out for Mandoto and Betafo (more than one subpopulation with seropositive rats). In Mandoto, significant correlations were found between differences in plague seroprevalence levels and genetic structure (*r* ^2^ = 12.0, *P* = 0.038) or geographic distance (*r* ^2^ = 16.7, *P* = 0.014). These spurious correlations were related to the subpopulation MAhiV characterized by the highest seroprevalence level of the area (Table 2; Figure 4A): when this subpopulation was removed from the analysis, partial Mantel tests were not significant (genetic structure: *r* ^2^ = 1.54, *P* = 0.47; geographic distance: *r* ^2^ = 0.4, *P* = 0.72). In Betafo, there was no significant correlation between differences in plague seroprevalence and genetic structure (*r* ^2^ = 1.2, *P* = 0.47) or geographic distance (*r* ^2^ = 0.1, *P* = 0.81). However, when the subpopulation BAtnV that is also characterized by the highest seroprevalence level of the area, was removed from the analysis, the correlation involving genetic structure became highly significant (*r* ^2^ = 42.6, *P* = 0.0001) (Figure 4B).

## Discussion

### Distribution of plague seroprevalence in rat populations

In Madagascar, the distribution of plague seroprevalence levels and reported plague cases was shown to differ between plateau and mountain areas in humans [11]. Our aim was to investigate whether the same pattern was observed in rat populations. In Ambositra and Moramanga, only one rat subpopulation per area had a positive seroprevalence for plague. This made it impossible to examine the relationship between topographic relief and seroprevalence distribution in these areas. The very small number of seropositive rats found in Ambositra (2 rats) was surprising, as this area reported the largest number of human cases during the 1990s [10]. Given the lifespan of wild *R. rattus* (about one year: [21]), this may indicate a low incidence of the disease during the year preceding sampling: indeed, six and five human cases were reported in the villages studied in Ambositra and Moramanga, respectively (Plague Laboratory database, Madagascan Ministry of Health). Alternatively, experimental infestations have shown that a substantial proportion of the rats from the highlands of Madagascar may be resistant to plague, remaining seronegative after exposure (even inoculated at high dose) [30]. For unknown reasons, these rats may be more frequent in Ambositra and Moramanga than elsewhere. Otherwise, plague seroprevalence data for rats were consistent with expectations in the other two areas studied: in Betafo (mountainous area), positive seroprevalence levels were clustered in the northwestern most subpopulations (BAln, BApg, BAtn, Table 2), whereas in Mandoto (plateau area), seroprevalence was positive for all subpopulations (Table 2). As for human plague seroprevalence data [11], this pattern may reflect differences in human lifestyles (due to a combination of historical and environmental factors) between Mandoto (more social interactions between villages) and Betafo (isolated villages), and hence more translocation of infected rats or fleas between villages in Mandoto than in Betafo. Alternatively, it may reflect differences in the spread of plague between reservoir subpopulations in mountains and those in plateau zones. Under this second hypothesis, we would expect a more marked population genetic structure for rats in the mountains than for those in plateau areas.

### Rat population genetics and topographic relief

In all areas, significant but weak genotypic differentiation of *R. rattus* subpopulations was observed over a relatively small spatial scale (between habitats in the same village, and between villages). Our results were globally consistent with the expectation of an increasing genetic structure of rat populations with topographic relief. Population genetic structure clearly increased from Moramanga (the flattest area) to Ambositra (the most mountainous area). No genetic isolation by distance was detected in Moramanga or Ambositra (Figure 3). This may reflect the occurrence of different processes, due to the differences in population genetic structure (see mean *F_ST_* in Table 2). In Moramanga, low levels of genetic structure may reflect large population sizes (as supported by *N_E_* estimates: Table 2), high levels of gene flow and random dispersal between subpopulations at the scale of this homogeneous landscape, mostly covered by rice fields. In Ambositra, high levels of genetic structure are suggestive of small populations (Table 2), low levels of gene flow and low levels of dispersal between subpopulations, which are isolated from each other even when geographically close. For instance, there was a high degree of genetic differentiation between AAklV and AAmpV (*F_ST_* = 0.10), two subpopulations that are only 3.5 km apart, but on different watersheds. Also, the highly differentiated ATitR subpopulation, despite its central location in the area studied, appears to be very small (*N_E_* = 5) and located in an isolated valley. Note however that the linkage disequilibrium method may provide biased downward estimates of *N_E_*, in case of pulse migration [55].

Population genetic structure estimates were however intermediate and not significantly different from each other (and from that of Ambositra) in Mandoto and Betafo (Table 2). Some indices, such as Structure results (Figure 2) or pairwise *F*_ST_ over large distances (Figure 3B, C), indicated that population structure was weaker in Mandoto, but we expected more clear-cut results given the differences in topographic relief between these two areas. In both areas, genetic differentiation increased with geographic distance, and IBD patterns were similar (Figure 3B, C). This suggests that our relief typology (mountainous area/plateau area) may have been too simplistic as concerns rat dispersal patterns. Rodent dispersal may be limited by more subtle physical properties of the surrounding environment, such as the fragmentation of favorable habitats (e.g., crops) by less favorable environments (e.g., steppes) or the occurrence of barriers, such as rivers [56], [57], [58], [59], which may not differ between Mandoto and Betafo. Indeed, further landscape genetic analyses would be helpful in order to describe in more details the influence of landscape on the population genetic structure of black rats.

### Rat population genetics and plague

Population genetic analyses indicated similar effective dispersal rates of rats in Betafo and Mandoto. Nevertheless, plague seroprevalence distribution was related to genetic structure in Betafo, but not in Mandoto (Figure 4). The role of rat dispersal regarding plague spread may be different in the two areas, reflecting their differences in human lifestyles. In Mandoto, the lack of concordance between seroprevalence data and genetic structure indicated that plague spread is not explained by the effective dispersal of the reservoir. The hypothesis that other mammal species disperse *Y. pestis*-infected fleas in this area, thereby counteracting the effect of rat population structure on disease spread (e.g., [14] in North American plague foci) appears unlikely, as trapping data showed the small mammals communities to be poorly diverse, largely dominated by *R. rattus* (98.5% of captures in Mandoto). Alternatively, rat population structure may play a less important role than human social interactions in determining the distribution of plague seroprevalence in rats and humans of this area. High levels of human movements in Mandoto may favor the translocation of *Y. pestis*-infected rodents and/or fleas between villages, thereby spreading plague in both the human and rat populations independently of their genetic or geographic proximity. There is ample evidence for the influence of human traffic on the spread of plague over larger distances, both in Madagascar and elsewhere [60]. Our results suggest that human-mediated *Y. pestis* transfers may also be important at a more local scale.

In the mountainous area of Betafo, the distribution of seroprevalence levels was clearly related to genetic structure when pairwise comparisons involving BAtnV are removed (Figure 4B). The positive correlation clearly suggests that rat dispersal has an influence on plague distribution in this area, except in BAtnV which was characterized by the highest plague seroprevalence level (Table 2). BAtnV had no spurious effects in IBD analyses. This village is located at immediate proximity of the main road of the area. BAtnV may thus be more related to distant human communities than the other villages of Betafo, which are all located at more than two kilometers from the asphalt road. As in Mandoto, few infected rodents, fleas, or humans (one human case reported in BAtnV in 2005; Plague Laboratory database, Madagascan Ministry of Health) may have reach BAtnV from a geographically distant location, and trigger an epidemics in local rats.

Empirical studies investigating the genetic consequences of infectious diseases in animal have shown that some populations lose genetic diversity [61], [62], [63], whereas others do not [64], [65]. Indeed, the impact of a decrease in population size on genetic diversity depends on the intensity of the disturbance, the length of time until recovery and the rate of recovery [66]. The positive seroprevalences obtained for some subpopulations indicated recent plague epizootics affecting the current generation of rats (Table 2). *F_I_*_S_ tended to be higher in subpopulations with positive plague seroprevalence in each area, especially in MAhiV and BAtnV (Table 2), but the relationship was significant only in Betafo. Thus, some indices indicated that plague epizootics affected the population genetic structure of the reservoir, but the evidence was not very strong. For instance, *N_E_* values were not significantly smaller in villages with a positive plague seroprevalence (Table 2). This suggests that plague epizootics have a small impact on population size, possibly because a substantial proportion of rats are resistant to the disease [30], [67], [68]. Stronger evidence for a causal relationship between plague and rat population genetics would require longitudinal surveys of rodent populations (e.g., [3]), with sampling before and after an epizootic to control for the large number of variables that may affect the estimated parameters.

### Conclusion

As expected, rat populations displayed a more marked genetic structure with increasing relief in highlands of Madagascar. Plague spread in rat and human populations can be partly related to the population genetic structure of the reservoir, but also to human social activities. Further improvements to our understanding of the consequences of landscape for plague distribution will require detailed analyses of the opportunities for movement open to the various participants in the plague cycle (human, rats, fleas), together with longitudinal surveys of plague seroprevalence in human and rat populations.
